# Comparison of Two α‐Synuclein Seed Amplification Assays for Discrimination of Parkinson Disease and Atypical Parkinsonism

**DOI:** 10.1002/mds.70017

**Published:** 2025-08-20

**Authors:** Marcello Rossi, Carly M. Farris, Simone Baiardi, Giulia Giannini, Franco Magliocchetti, Luisa Sambati, Yihua Ma, Erica Vittoriosi, Giovanna Calandra‐Buonaura, Luis Concha‐Marambio, Piero Parchi

**Affiliations:** ^1^ IRCCS Istituto delle Scienze Neurologiche di Bologna Bologna Italy; ^2^ Amprion, R&D Unit San Diego California USA; ^3^ Department of Biomedical and Neuromotor Sciences University of Bologna Bologna Italy

**Keywords:** seed amplification assay, biomarker, CSF, RT‐QuIC, synucleinopathies, prion disease

## Abstract

**Background:**

Seed amplification assays (SAAs) for misfolded α‐synuclein (syn) have shown inconsistent results in multiple system atrophy (MSA).

**Objective:**

The objective of this study was to compare a novel syn SAA (synSAA) that distinguishes between Lewy body disease (LBD) and MSA syn‐seeds (Amprion‐SAA) with an LBD‐specific synSAA (IRCCS Istituto delle Scienze Neurologiche di Bologna [ISNB]‐SAA).

**Methods:**

We applied both assays to cerebrospinal fluid samples from 114 patients with MSA, 49 patients with Parkinson disease (PD), 40 patients with progressive supranuclear palsy (PSP), and 46 controls.

**Results:**

Amprion‐SAA detected type 2 (“MSA‐type”) syn‐seeds in 101 (88.6%) MSA, 3 (6.1%) PD, 4 (10.0%) PSP, and 6 (13.0%) control participants, and type 1 (“LBD‐type”) syn‐seeds in 39 (79.6%) PD, 3 (2.6%) MSA, and 1 (2.5%) PSP participant. ISNB‐SAA detected LBD‐specific syn‐seeds in 40 (81.6%) PD, 4 (3.5%) MSA, and none of the PSP or control participants.

**Conclusions:**

Amprion‐SAA, performed at ISNB, uniquely discriminated MSA from both PD and PSP participants with good accuracy. However, it showed lower specificity than ISNB‐SAA, primarily related to the type 2 profile. © 2025 The Author(s). *Movement Disorders* published by Wiley Periodicals LLC on behalf of International Parkinson and Movement Disorder Society.

## Introduction

Parkinson disease (PD) and multiple system atrophy (MSA) are synucleinopathies that respectively present Lewy body pathology and glial cytoplasmic inclusions as hallmark pathological findings. PD, MSA, and progressive supranuclear palsy (PSP), another parkinsonian disorder with tau rather than α‐synuclein (syn) pathology, are difficult to differentiate clinically, especially in the early stages. Because PSP and MSA progress more rapidly and show a poor response to levodopa (l‐dopa), accurate diagnosis is necessary for prognosis and clinical trials. Current diagnostic criteria for parkinsonian disorders require clinical findings, multiple diagnostic investigations, and follow‐up to reach an accurate disease identification, limiting the early diagnosis.^1^, ^2^, ^3^ In this context, the detection of misfolded syn aggregates (syn‐seeds) by seed amplification assay (SAA) has provided a robust biomarker for PD^4^ but also raised the need to distinguish between synucleinopathies. Although initial synSAA results for MSA have been inconsistent, with sensitivities ranging from 6% to 85%,^4^ a novel synSAA has shown high performance in detecting and differentiating Lewy body disease (LBD) (type 1 syn‐seeds) and MSA (type 2 syn‐seeds).^5^ In this study, we implemented this novel synSAA and analyzed a large single‐center cohort of well‐characterized MSA patients with significant follow‐up and thorough diagnostic assessment. As a second aim, we compared the performance of this novel synSAA protocol with an internally validated synSAA specific for LBD in patients with MSA, PD, and PSP and controls.

## Subjects and Methods

### Participants

All participants were recruited at the IRCCS Istituto delle Scienze Neurologiche di Bologna (ISNB) between 2010 and 2023.

Selection criteria were limited to the following: (1) the availability of cerebrospinal fluid (CSF) for analyses; and (2) a “clinically established,” “clinically probable,” or “neuropathologically established” diagnosis according to MDS diagnostic criteria for MSA^1^ and PD,^2^ or a probable or possible diagnosis for PSP^3^ (Supporting Information Table S1). MSA and PSP patients were consecutively enrolled; in contrast, PD participants consisted of a convenience cohort enriched with cases synSAA^−^ or showing low seeding activity. Details regarding the number of examinations, diagnostic assessment, level of diagnostic certainty, and disease phenotype (MSA and PSP) are provided in the Supporting Information and Tables S1–S4. The control group included subjects aged <50 years (mean 21.5 ± 12.8 years) and lacking any clinical and neuroradiological evidence of disease affecting the nervous system (eg, patients with chronic headaches or mild psychiatric symptoms).

The study was approved by the local Ethics Committee (approval numbers AVEC 09070, 17093, 18025, and 18027), and participants provided written informed consent.

### synSAA

All synSAA analyses were performed at ISNB. CSF samples were analyzed using two protocols: the synSAA originally developed in Caughey's lab^6^ and adapted at ISNB,^7^ and the novel synSAA developed by Amprion.^5^

With the ISNB‐synSAA protocol (see Supporting Information),^8^ samples were run in quadruplicate and deemed positive when at least three of the four replicates exceeded the threshold, calculated as 30% of the median maximum fluorescence (F_max_) of the positive control replicates. When one or two replicates crossed this threshold, the sample was run up to three times. With 12 loaded replicates, the samples were considered positive if they exhibited seeding activity in four or more.

Amprion provided all the reagents and materials for the novel synSAA protocol (see Supporting Information).^5^ All samples were analyzed in triplicate. A single operator performed all analyses using an Atto425‐calibrated FLUOstar Omega plate reader. Replicates were classified according to their F_max_ as type 1 (F_max_ ≥ 45,000 relative fluorescence units [RFUs]), type 2 (F_max_ < 45,000 and ≥3,000 RFUs), or negative (F_max_ < 3,000 RFUs). Samples with three type 1 replicates were deemed synSAA^+^ type 1. Samples with two or three type 2 replicates were deemed synSAA^+^ type 2. Samples with two type 1 and one type 2 replicate were deemed synSAA^+^ undetermined. Samples with two or three negative replicates were deemed synSAA negative (synSAA^−^). Other cases were deemed inconclusive. Undetermined/Inconclusive results were retested in a further independent synSAA analysis before being deemed conclusive.

## Data Analysis

Sensitivity was calculated as the proportion of synSAA^+^ cases within the groups diagnosed with a given synucleinopathy (PD or MSA). For the ISNB assay, specificity was calculated as 1 minus the proportion of synSAA^+^ cases among participants without a diagnosis of PD. For the novel synSAA, specificity was also calculated separately for the type 1 (LBD) and type 2 (MSA) outcomes. Specificity for LBD was 1 minus the proportion of type 1 results in MSA, PSP, and control cases. Specificity for MSA was 1 minus the proportion of type 2 results in patients with PD, PSP and controls. Graphs and statistical analysis were performed with GraphPad.

## Results

### Performance of the Novel synSAA

**FIG. 1:**
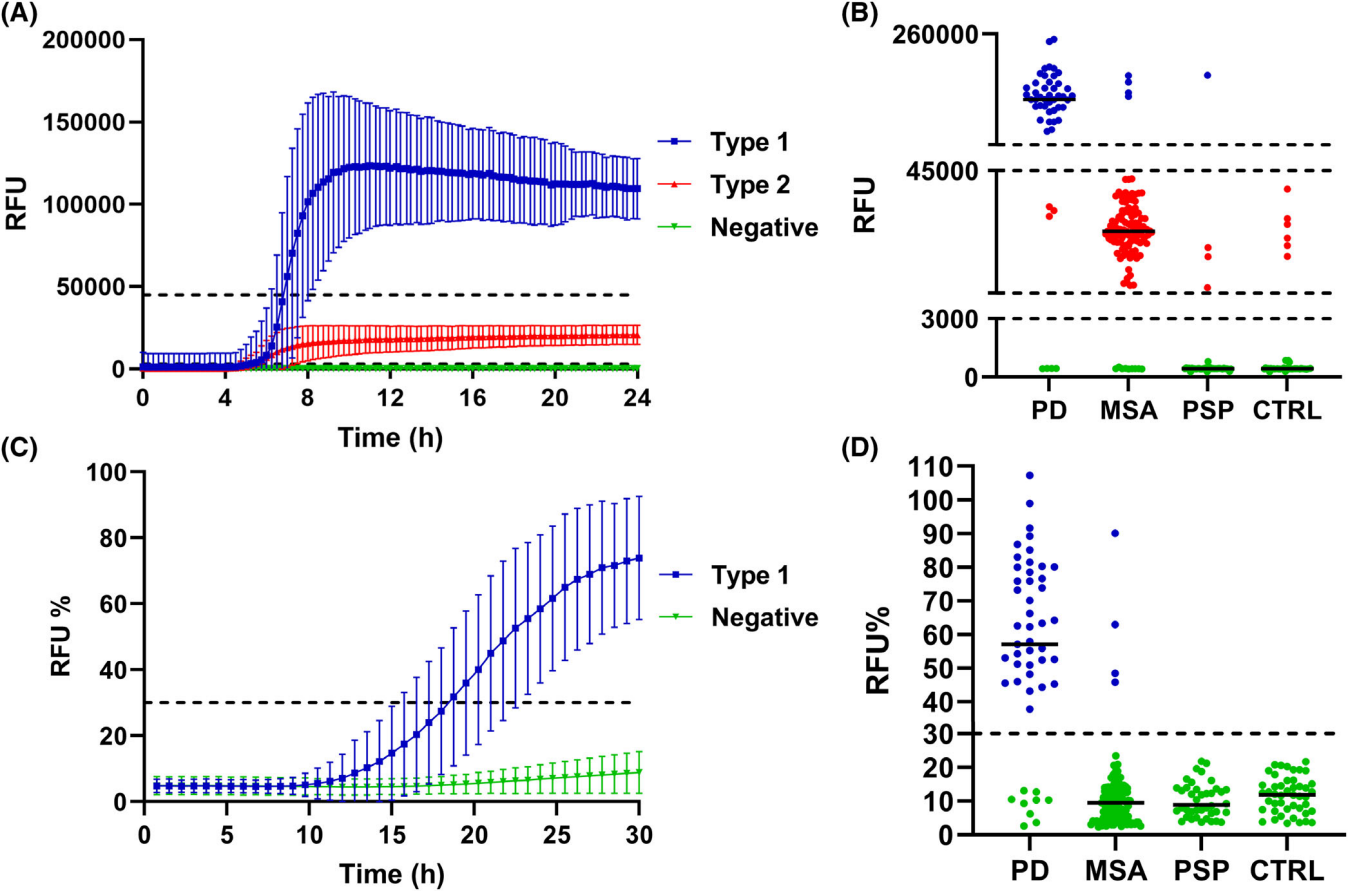
Graphic representation of the results obtained with the Amprion‐ and ISNB‐synSAAs. Panels **A** and **C** illustrate the kinetic profiles of the fluorescence signals generated by the two assays. (**A**) Three distinct profiles (type 1, type 2, and negative) can be distinguished with the Amprion‐synSAA based on two distinct thresholds. (**B**) In contrast, only two profiles (positive vs. negative) based on a single signal threshold characterize the ISNB‐synSAA. Values are expressed as the average of all replicates belonging to each kinetic profile ± standard deviation. (**B** and **D**) The maximum fluorescence (F_max_) of the cases categorized by study cohort. F_max_ values are expressed as the median of the sample replicates. The black lines describe the median value of each diagnostic group. ISNB‐SAA results (**C**, **D**) are normalized as described in Supporting Information Materials and Methods and shown in RFUs (%). Among the samples repeated three times with ISNB‐SAA after the initial unclear result, the first negative result obtained was selected to be represented in the graph. CTRL, control; MSA, multiple system atrophy; PD, Parkinson disease; PSP, progressive supranuclear palsy; RFUs, relative fluorescence units. [Color figure can be viewed at wileyonlinelibrary.com]

Of the 114 patients with MSA, 101 (88.6%) were synSAA^+^ type 2, including all those with a definite neuropathological diagnosis (n = 4, 100%). Of the other 13, 3 (2.6%) were synSAA^+^ type 1, 1 (0.9%) was synSAA^+^ undetermined, and 9 (7.9%) were synSAA^−^ (Fig. 1, Table 1). In contrast, among the 49 PD participants, 39 (79.6%) showed synSAA^+^ type 1, 3 (6.1%) were synSAA^+^ type 2, 3 (6.1%) were synSAA^+^ undetermined, and 4 (8.2%) were synSAA^−^.

**TABLE 1:** Overall performance of Amprion‐ and ISNB‐synSAAs

	Amprion‐SAA		ISNB‐SAA
Diagnostic groups	Overall syn seeding activity, +/−		
PD	45/4		40/9
MSA	105/9		4/110
PSP	4/35 and 1 inconclusive		0/40
Controls	6/40		0/46
Diagnostic performance (95% CI)			
Sensitivity			
PD	91.8% (80.4–97.7)		81.6% (68.0–91.2)
MSA	92.1% (85.5–96.33)		–
Specificity			
MSA	–		96.5% (91.3–99.0)
PSP	90.0% (76.3–97.2)		100% (91.2–100.0)
Controls	87.0% (73.7–95.0)		100% (92.3–100.0)
Diagnostic groups	Kinetic profile type 1 (LBD‐like), +/−	Kinetic profile type 2 (MSA‐like), +/−	NA
PD^a^	39/10	3/46	–
MSA^a^	3/111	101/13	–
PSP	1/39	3/37	–
Controls	0/46	6/40	–
Diagnostic performance (95% CI)			
Sensitivity, kinetic profile type 1 for PD	79.6% (65.7–89.8)	–	–
Specificity, kinetic profile type 1			
PD vs. MSA	97.4% (92.5–99.4)	–	–
PD vs. PSP	97.5% (86.8–99.9)	–	–
PD vs. controls	100% (92.3–100.0)	–	–
Sensitivity, kinetic profile type 2 for MSA	–	88.6% (81.3–93.8)	–
Specificity, kinetic profile type 2			
MSA vs. PD	–	93.9% (83.1–98.7)	–
MSA vs. PSP	–	92.5% (79.6–98.4)	–
MSA vs. controls	–	87.0% (73.7–95.1)	–

^a^ The kinetic profile was synSAA^+^ undetermined in three PD and one MSA case.

Abbreviations: ISNB, IRCCS Istituto delle Scienze Neurologiche di Bologna; synSAA, α‐synuclein seed amplification assay; SAA, seed amplification assay; PD, Parkinson disease; MSA, multiple system atrophy; PSP, progressive supranuclear palsy; LBD, Lewy body disease; CI, confidence interval; NA, not applicable.

In the other groups, four PSPs (10.0%) and six controls (13.0%) showed seeding activity. They were all type 2 except for two PSP participants (one type 1 and one inconclusive result).

The overall sensitivity for syn seeding activity was 92.1% in MSA and 91.8% in PD, with a specificity of 90.0% against PSP and 87.0% against controls. The sensitivity of the type 2 profile in MSA was 88.6%, with a 93.9% specificity against PD. The sensitivity for LBD (type 1 profile) in PD patients was 79.6%, with a specificity of 97.4% against MSA, 97.5% against PSP, and 100% against controls (Table 1).

There were no significant differences in follow‐up duration, number of performed diagnostic investigations, and classification between participants with congruent (type 2 in MSA and negative SAA in PSP) and unexpected (all other outcomes) results in MSA and PSP groups (Supporting Information Tables S1–S4).

One CSF sample from the MSA, PD, and control groups was replicated six times on different plates to verify the assay's repeatability. All runs showed synSAA^+^ type 1 and type 2, respectively, for the PD and MSA samples, whereas the control CSF was always synSAA^−^.

## Comparison of the Novel synSAA and ISNB‐synSAA Diagnostic Performance

As a secondary aim, we compared the performance of the novel synSAA and the ISNB‐synSAA, which was also validated in patients undergoing postmortem brain examination.^9^ The main difference between the two is that the ISNB‐SAA shows no syn seeding activity in MSA patients' CSF or brain samples. Therefore, a positive fluorescence curve profile is highly specific for LBD‐associated syn.

The ISNB‐synSAA showed positive syn seeding activity in 40 (81.6%) patients with PD and four (3.5%) patients with MSA, whereas no seeding activity was detected in PSP and control CSF samples (Fig. 1). The sensitivity for LBD in patients with PD was 81.6%, with a specificity of 96.5% against MSA, 100% against PSP, and 100% against controls.

Overall, 35 patients with PD showed a positivity for LBD with both assays (Supporting Information Table S5). Among the nine negative ones by ISNB‐synSAA, five showed CSF seeding activity using the novel synSAA, including four type 1 and one type 2 profile. In these participants, the level of diagnostic certainty was “clinically established”, the disease duration at lumbar puncture was 1 to 3 years, and the follow‐up was 4 to 15 years.

On the other hand, five PD patients positive with the ISNB assay yielded a positive‐undetermined (n = 3) or type 2 positive (n = 2) result with the Amprion assay (Supporting Information Tables S2 and S5). All had a “clinically established” level of diagnostic certainty, with a clinical follow‐up of 4 to 10 years.

Regarding the four MSA cases with a positive result by ISNB‐synSAA, two were type 2, one type 1, and one was synSAA^+^ undetermined by the novel synSAA (Supporting Information Tables S2 and S5).

## Discussion

We implemented the novel synSAA conditions recently published by Ma and collaborators^5^ in ISNB to determine whether the identification of type 2 syn‐seeds could be replicated in a distinctive large cohort of patients with MSA and when the assay is performed in an independent laboratory. The assay detected type 2 syn‐seeds in 88.6% (and 92.1% seeding activity overall) of patients with a clinical or neuropathological diagnosis of MSA, whereas 79.6% of the synSAA^+^ clinically defined PD cases presented type 1 syn‐seeds. Moreover, 91.8% of patients with PD showed syn seeding activity overall. Specificity for type 2 syn‐seeds reached 92.5% for PSP and 87.0% for controls, comparable with the 90.0% previously reported for the non‐synucleinopathy group.^5^ Given the mean young age of the controls we selected to exclude the possibility of age‐related incidental LB or MSA pathology, our results suggest that the type 2 profile for MSA is less specific than the type 1 for LBD. Indeed, the specificity of the type 1 profile against patients with PSP and controls was almost complete and comparable with that of the ISNB assay, which detects only LBD seeds.

These results show the capacity of the novel synSAA to recognize MSA as a distinct type of synucleinopathy, distinguishable from both LBD and non‐synucleinopathies manifesting a progressive parkinsonism. The MSA patient cohort analyzed in this study presented very similar results (88.6% sensitivity) as the research cohorts reported by Ma and colleagues^5^ (85%, 83%), reflecting the consistency of the results when including patients with deep clinical phenotyping and clinical follow‐up, recruited in reference centers for MSA, such as the ISNB.^10^, ^11^

Despite the focus on MSA, we also compared the sensitivity for LBD. To this end, we aimed to increase the probability of detecting differences in accuracy between the two assays by selecting a convenience cohort enriched with samples from patients with a clinical diagnosis of PD who showed either no seeds or low seeding activity with the ISNB assay.^12^ The sensitivity for LBD (ie, positive seeding activity for the ISNB assay and type 1 profile for the novel assay) was nearly 80% for both assays. However, when all positive profiles were considered (ie, type 1, positive‐undetermined, and type 2), the Amprion‐synSAA showed slightly higher sensitivity for syn‐seeds in PD than the ISNB‐synSAA.

In conclusion, this study confirms the high performance of the novel Amprion‐synSAA for detecting and differentiating syn‐seeds in LBD and MSA. The unique capability to generate a distinctive kinetic profile in LBD and MSA represents an added value of this assay. In contrast, the multiple reading outcomes (type 1, type 2, and positive‐undetermined) and the incomplete specificity of the type 2 profile may introduce some uncertainty in the interpretation of the results in some cases. Indeed, the dichotomous outcome (positive vs. negative for LBD) and the high specificity for LBD of the ISNB assay provide a message with unambiguous interpretation in the case of a positive outcome. However, the latter does not help distinguish between MSA and other non‐LBD parkinsonism. Thus, the two synSAA protocols represent valid complementary alternatives that might help resolve inconclusive cases.

## Author Roles

(1) Research project: A. Conception, B. Organization, C. Execution;

(2) Patient recruitment: A. Physical examination, B. Documentation;

(3) Data analysis: A. Design, B. Execution, C. Review and critique;

(4) Manuscript preparation: A. Writing of the first draft, B. Review and critique.

M.R.: 1B, 1C, 3A, 3B, 3C, 4A, 4B

C.M.F.: 1B, 1C

S.B.: 1C, 2A, 2B, 3A, 3B, 3C, 4B

G.G.: 2A, 2B, 3C

F.M.: 1C

L.S.: 2A, 2B, 3C

Y.M.: 1B, 1C

E.V.: 1C

G.C.‐B.: 2A, 2B, 3C

L.C.‐M.: 1A, 1B, 1C, 3C, 4A, 4B

P.P.: 1A, 1B, 2B, 3A, 3B, 3C, 4A, 4B

## Full Financial Disclosures for the Previous 12 Months

P.P. has received funding from the Italian Ministero della Salute and Ministero della Ricerca Scientifica. M.R. was funded from the Carisbo Foundation. L.C.‐M., C.M.F., and Y.M. are employees of Amprion Inc. and hold stock options. L.C.‐M., C.M.F., and Y.M. are named inventors of several patents and pending patent applications related to the α‐synuclein seed amplification assay. These patents and pending applications are either co‐owned by Amprion with UT Health and exclusively licensed by Amprion Inc. or are owned solely by Amprion. None of the other authors have any financial disclosures.

## Supporting information


**Data S1.** Supporting Information.

**Table S1.** Demographic and clinical data.
**Table S2.** Level of diagnostic certainty, demographic and clinical features of MSA patients showing either discrepant results between ISNB and Amprion SynSAA assays, or unexpected results.
**Table S3.** Level of diagnostic certainly, demographic and clinical features of PSP patients showing discrepant results between ISNB‐ and Amprion‐SynSAA assays.
**Table S4.** Diagnostic investigations in MSA cases with positive or discordant SAA results.

**Table S5.** Comparison of the results with the two assays in each diagnostic group and participant.

## Data Availability

The data that support the findings of this study are available from the corresponding author upon reasonable request.
