# Supplementary material for: Comparison of Two α‐Synuclein Seed Amplification Assays for Discrimination of Parkinson Disease and Atypical Parkinsonism
Source: Mov Disord. 2025 Aug 20;40(11):2504–9. doi: 10.1002/mds.70017 (PMC12661618; doi:10.1002/mds.70017)
Supplement: Supplementary file 1 — Data S1. Supporting Information. [file MDS-40-2504-s003.docx]

**Comparison of two α-synuclein SAAs for discrimination of Parkinson's disease and atypical parkinsonism**

Marcello Rossi PhD, Carly M Farris MSc, Simone Baiardi MD, PhD, Giulia Giannini MD, PhD, Franco Magliocchetti MSc, Luisa Sambati MD, PhD, Yihua Ma MSc, Erica Vittoriosi MSc, Giovanna Calandra-Buonaura MD, PhD, Luis Concha-Marambio PhD, Piero Parchi MD, PhD

**Supplementary Material and Methods**

**Patients and Methods**

The MSA patients included in the study belonged to a well-characterised retrospective and prospective cohort enrolled at IRCCS Institute of Neurological Sciences of Bologna (MSA-BO cohort). In the prospective cohort, patients were evaluated every 6 months from enrolment throughout the follow-up period. In the retrospective cohort, we included patients referred between 2005 and 2017 with a clinical diagnosis of MSA who were evaluated at least once a year during the disease course. Three neurologists, experts in movement disorders, independently confirmed the diagnosis of MSA based on the data available at the last follow-up evaluation.

Besides a thorough clinical investigation, the following instrumental/laboratory tests were analysed when available: 1) brain MRI or CT (if MRI was not possible); 2) neuropsychological evaluation; 3) head-up tilt test and other cardiovascular reflex tests; 4) effect of levodopa (if applicable) assessed by a) a standardized oral levodopa kinetic-dynamic test, b) improvement of part III of the Unified Parkinson’s Disease Rating Scale after increasing levodopa up to 1 g/die; 5) cardiac ^123^I-metaiodobenzylguanidin (MIBG)-SPECT; 6) cerebral ^123^I-ioflupane-SPECT; 7) determination of CSF NfL levels. Instrumental and laboratory tests were performed during the disease course if required for differential diagnosis or to elucidate specific conditions suspected by history or examination^1-8^. Regarding the number of instrumental/laboratory tests, brain MRI was available in 103/114 patients, videopolysomnography (VPSG) in 100/114, neuropsychological evaluation in 75/114, cardiovascular reflex test in 103/114, cardiac MIBG-SPECT in 59/114, cerebral 123I-ioflupane-SPECT in 88/114. VPSG was performed according to the protocol described in Vetrugno et al, 2007)^9^. RBD was confirmed by VPSG and diagnosed according to American Academy of Sleep Medicine criteria^11^. PD participants were selected after a screening at ISNB for α-syn seeding activity. They were classified as negative (9 cases), “low” seeders (10 cases) or “high” seeders (30 cases) according to Mammana et. al.^10^ Briefly, the low seeding cases gave 3-5 positive replicates (out of 8), whereas the “high” seeding group included cases yielding ≥7 positive replicates (out of 8) in the ISNB-synSAA.

Further clinical and demographic information, as well as levels of diagnostic certainty in each diagnostic group, are reported in Table S1.

**CSF collection and analyses**

CSF was obtained by lumbar puncture at the L3/L4 or L4/L5 levels, aliquoted within 2 hours and stored in polypropylene tubes at -80 °C until analyses.

In all samples, we measured neurofilament light chain (NfL) using a validated commercial enzyme-linked immunosorbent assay (NfL ELISA kit, IBL, Hamburg, Germany). The mean intra- and inter-assay coefficients of variation (CVs) were 4% and 10%^12^.

**SynSAA**

The ISNB-synSAA was performed in black 96-well plates with a clear bottom (Nalgene Nunc International) pre-loaded with six 0.8 mm silica beads (OPS Diagnostics) per well. CSF samples were thawed and vortexed for 10 seconds before use. Fifteen µL of CSF were added as seed to trigger the reaction in 85 µL of a mixture containing 40 mM PB, pH 8.0, 170 mM NaCl, 10 µM thioflavin-T (ThT), 0.0015% sodium dodecyl sulfate (SDS), and 0.1 mg/ml of filtered (100 kDa MWCO filters) recombinant α-Syn produced in-house^13^. The plate was sealed with a plate sealer film (Nalgene Nunc International) and incubated in a FLUOstar Omega (BMG Labtech) plate reader at 42 °C with intermittent double orbital shaking at 400 rpm for one minute, followed by a 1-minute rest. ThT fluorescence measurements were taken for 30 hours every 45 minutes using 450 nm excitation and 480 nm emission filters. Samples were run in quadruplicates and deemed positive when at least three of four replicates reached the threshold, calculated as 30% of the median of the highest fluorescence (Imax) of the positive control replicates. When one or two replicates crossed this threshold, the sample was run up to three times. With a total of 12 replicates, the samples were considered positive if they showed seeding activity in four or more replicates.

The novel synSAA developed by Amprion was performed in a final 100μL reaction mixture, consisting of 100mM PIPES pH 6.50 (Sigma, cat#80635), 500mM NaCl (Lonza, cat#51202), 10μM ThT (Sigma, cat#T3516), 0.1% sarkosyl (Sigma, cat#61747-13 100ML), 0.3mg/mL rec-αSyn (Amprion, cat#S2020), and 40μL CSF. Clear bottom plates (Greiner, cat#655906) were used with 2x 3mm borosilicate beads per well (Pyrex). Beads were added first, followed by the untreated CSF samples (previously vortexed for 10 seconds), and then the master mix, including unfiltered substrates. Plate and CSF were maintained on ice for the entire loading process. The plate was sealed with a plate sealer film (Nalgene Nunc International). Plates were evaluated for 24 hours at 42°C with intermittent orbital shaking (800rpm) for 1 minute every 15 minutes. Fluorescence was read every cycle using fluorescence-calibrated FLUOstar Omegas (BMG), with filters set at 450 nm excitation and 480 nm emission.

All samples were analyzed in adjacent triplicates. We identified replicates with high fluorescence (≥45,000) RFU as Type1, intermediate fluorescence (<45,000 and ≥3,000) as Type2, and those with fluorescence indistinguishable from the background (<3000 RFU) as negative. Maximum fluorescence (Fmax) from three replicates was combined in a determination criterion to generate a dual output for each sample; the first output determines the detection/non-detection of syn-seeds, and the second output determines the type of syn-seeds present in synSAA-positive (synSAA+) samples. Briefly, samples with three Type1 replicates (Fmax≥45,000RFU) were deemed synSAA+ Type1. Samples with two or three Type2 replicates (Fmax between 3,000RFU and 45,000RFU) were deemed synSAA+ Type2. Samples with two Type1 and one Type2 replicates were deemed synSAA+ undetermined. Samples with two or three negative (Fmax<3,000RFU) replicates were deemed synSAA 2 negative (synSAA-). Other cases were deemed inconclusive and undetermined. Inconclusive and undetermined results triggered a sample retest if the sample volume allowed.

For samples generating Inconclusive and/or Undetermined results, we repeated the analysis in triplicate in a subsequent run to obtain conclusive final results. If the re-run produced Inconclusive and/or Undetermined results again, we deemed the sample Inconclusive/Undetermined as the final result.

**Neuropathologic Examination**

In 4 patients (MSA group), neuropathological examination was performed at the Neuropathology Laboratory of IRCCS Institute of Neurological Sciences of Bologna using standardized procedures as described^14^. Seven μm-thick sections from each block were stained with hematoxylin–eosin for screening. Also, immunohistochemistry with antibodies specific for αSyn (LB509, dilution 1:100, Thermo Fisher Scientific, and KM51, dilution 1:500, Novocastra) was applied to all cases across several brain regions, following established consensus criteria^15^. An experienced neuropathologist (PP) formulated the final diagnosis in accordance with consensus criteria^15^.

**References**

1. Calandra-Buonaura G, Guaraldi P, Sambati L, et al. Multiple system atrophy with prolonged survival: is late onset of dysautonomia the clue? Neurol Sci. 2013;34(10):1875-8.
2. Calandra-Buonaura G, Doria A, Lopane G, et al. Pharmacodynamics of a low subacute levodopa dose helps distinguish between multiple system atrophy with predominant Parkinsonism and Parkinson's disease. J Neurol. 2016;263(2):250-256.
3. Giannini G, Calandra-Buonaura G, Mastrolilli F, et al. Early stridor onset and stridor treatment predict survival in 136 patients with MSA. Neurology. 2016;87(13):1375-83.
4. Giannini G, Mastrangelo V, Provini F, et al. Progression and prognosis in multiple system atrophy presenting with REM behavior disorder. Neurology. 2020;94(17):e1828-e1834.
5. Giannini G, Provini F, Cani I, et al. Tracheostomy is associated with increased survival in Multiple System Atrophy patients with stridor. Eur J Neurol. 2022;29(8):2232-2240.
6. Giannini G, Baldelli L, Provini F, et al. Early onset sleep disorders predict severity, progression and death in multiple system atrophy. J Neurol. 2025;272(3):239.
7. Baschieri F, Calandra-Buonaura G, Doria A, et al. Cardiovascular autonomic testing performed with a new integrated instrumental approach is useful in differentiating MSA-P from PD at an early stage. Parkinsonism Relat Disord. 2015;21(5):477-82.
8. Quadalti C, Calandra-Buonaura G, Baiardi S, et al. Neurofilament light chain and α-synuclein RT-QuIC as differential diagnostic biomarkers in parkinsonisms and related syndromes. NPJ Parkinsons Dis. 2021;7(1):93.
9. Vetrugno R, Liguori R, Cortelli P, et al. Sleep-related stridor due to dystonic vocal cord motion and neurogenic tachypnea/tachycardia in multiple system atrophy. Mov Disord. 2007 Apr 15;22(5):673-8. doi: 10.1002/mds.21384. PMID: 17266093.
10. American Academy of Sleep Medicine criteria International Classification of Sleep Disorders - Third Edition (ICSD-3). Darien, IL: American Academy of Sleep Medicine.
11. Mammana A, Baiardi S, Rossi M, et al. Improving protocols for α-synuclein seed amplification assays: analysis of preanalytical and analytical variables and identification of candidate parameters for seed quantification. Clin Chem Lab Med. 2024;62(10):2001-2010.
12. Baiardi S, Rossi M, Giannini G, et al. Head-to-head comparison of four cerebrospinal fluid and three plasma neurofilament light chain assays in Parkinsonism. NPJ Parkinsons Dis. 2025;11(1):98.
13. Rossi M, Candelise N, Baiardi S, et al. Ultrasensitive RT-QuIC assay with high sensitivity and specificity for Lewy body-associated synucleinopathies. Acta Neuropathol 2020;140(1):49-62.
14. Lattanzio F, Abu-Rumeileh S, Franceschini A, et al. Prion-specific and surrogate CSF biomarkers in Creutzfeldt-Jakob disease: diagnostic accuracy in relation to molecular subtypes and analysis of neuropathological correlates of p-tau and Aβ42 levels. Acta Neuropathol. 2017;133:559–578.
15. Trojanowski JQ, Revesz T; Neuropathology Working Group on MSA. Proposed neuropathological criteria for the post mortem diagnosis of multiple system atrophy. Neuropathol Appl Neurobiol. 2007;33(6):615-20.
