# Supplementary material for: Comparison of Two α‐Synuclein Seed Amplification Assays for Discrimination of Parkinson Disease and Atypical Parkinsonism
Source: Mov Disord. 2025 Aug 20;40(11):2504–9. doi: 10.1002/mds.70017 (PMC12661618; doi:10.1002/mds.70017)
Supplement: Supplementary file 2 — Table S1. Demographic and clinical data. Table S2. Level of diagnostic certainty, demographic and clinical features of MSA patients showing either discrepant results between ISNB and Amprion SynSAA assays, or unexpected results. Table S3. Level of diagnostic certainly, demographic and clinical features of PSP patients showing discrepant results between ISNB‐ and Amprion‐SynSAA assays. Table S4. Diagnostic investigations in MSA cases with positive or discordant SAA results. [file MDS-40-2504-s002.docx]

|  | **MSA (n=114)** | **PD (n=49)** | **PSP (n=40)** |
| --- | --- | --- | --- |
|  | Clinically probable: 24 (21.1%); 18 MSA-C, 6 MSA-P  Clinically established: 86 (75.4%); 34 MSA-C, 52 MSA-P  Neuropathologically established: 4 (3.5%); 1 MSA-C, 3 MSA-P | Clinically Probable: 7 (14.3%)  Clinically Established: 42 (85.1%) | Possible: 4 (10.0%)  Probable: 36 (90.0%) |
| Age at CSF (y) | 62.4 ± 8.1  Range: 41-79 | 62.1 ± 8.9  Range: 41-80 | 73.0 ± 7.7  Range: 51-89 |
| Disease Duration at CSF (y) | 4.2 ± 2.3  Range: 1-13 | 5.2 ± 4.8  Range: 1-27 | 4.7 ± 2.6  Range: 1-13 |
| Total follow-up duration from disease onset (y) | 6.9 ± 2.9  Range: 1-17 | 8.4 ± 4.9  Range: 2-28 | 6.4 ± 2.4  Range: 3-14 |
| Phenotype C/P | 53 (46.5%) / 61 (53.5%) | n.a. | n.a. |
| CSF NfL, pg/ml | 3150.0 ± 1197.0 | 676.3 ± 385.4 | 2043.0 ± 605.1 |

**Table S1**. Demographic and clinical data

Legend: y = years, C = cerebellar, P = parkinsonism, NfL = Neurofilament light chain, n.a. = not applicable

**Table S2**. Level of diagnostic certainty, demographic and clinical features of MSA patients showing either discrepant results between ISNB and Amprion SynSAA assays, or unexpected results.

| **ID** | **Level of certainty of MSA diagnosis at last follow-up*** | **Phenotype**** | **Sex** | **Age at onset (years)** | **Time onset-lumbar puncture**  **(years)** | **Time onset-last follow-up** (years)** | **Diagnostic Investigations** | **Less frequent features for MSA***** | **ISNB-SynSAA result** | **Amprion-SynSAA result** |
| --- | --- | --- | --- | --- | --- | --- | --- | --- | --- | --- |
| ***Discrepant SynSAA results*** | | | | | | | | | | |
| MSA-17 | Clinically Established | P | M | 69 | 2 | 5 | 1, 2, 3, 4, 5, 6, 7 | MIBG abnormal | Negative | Positive-LBD-like |
| MSA-63 | Clinically Established | P | F | 64 | 9 | 12 | 1, 2, 3, 4, 5, 6, 7 | None | Positive | Positive-MSA-like |
| MSA-71 | Clinically Probable | C | M | 68 | 1 | 2 | 2, 4, 5, 6, 7 | Cognitive impairment | Positive | Positive-Undetermined |
| MSA-111 | Clinically Established | P | M | 49 | 8 | 9 | 1, 2, 4, 5, 6, 7 | None | Positive | Positive-MSA-like |
| MSA-112 | Clinically Established | C | F | 70 | 7 | 7 | 1, 2, 4, 5, 6, 7 | None | Negative | Positive-LBD-like |
| ***Unexpected SynSAA results*** | | | | | | | | | | |
| MSA-18 | Clinically Probable | P | M | 65 | 2 | 9 | 1, 2, 3, 4, 5, 6, 7 | None | Negative | Negative |
| MSA-20 | Clinically Probable | C | M | 58 | 2 | 10 | 1, 2, 3, 4, 5, 6, 7 | None | Negative | Negative |
| MSA-31 | Clinically Probable | C | F | 57 | 4 | 10 | 1, 2, 3, 4, 5, 6, 7 | None | Negative | Negative |
| MSA-41 | Clinically Established | P | F | 57 | 3 | 7 | 1, 4, 5, 6, 7 | None | Negative | Negative |
| MSA-61 | Clinically Established | C | M | 51 | 2 | 8 | 1, 2, 5, 6, 7 | None | Negative | Negative |
| MSA-84 | Clinically Established | C | F | 52 | 8 | 11 | 1, 2, 4, 5, 6, 7 | None | Negative | Negative |
| MSA-86 | Clinically Probable | C | M | 61 | 4 | 4 | 1, 2, 4, 6, 7 | Cognitive impairment | Positive | Positive-LBD-like |
| MSA-96 | Clinically Established | C | F | 56 | 3 | 5 | 1, 2, 4, 5, 6, 7 | None | Negative | Negative |
| MSA-100 | Clinically Established | P | F | 72 | 3 | 5 | 1, 2, 4, 5, 6, 7 | None | Negative | Negative |
| MSA-110 | Clinically Established | P | F | 61 | 4 | 5 | 1, 2, 3, 4, 5, 6, 7 | None | Negative | Negative |

*According to Wenning et al. 2022 *Movement Disorder* (ref. 1 in the main text). Diagnostic investigation: (1) brain magnetic resonance imaging; (2) Cerebral ^123^I-ioflupane-SPECT; (3) Cardiac ^123^I-MIBG-scintigraphy; (4) neuropsychological tests; (5) videopolysomnography; (6) cardiovascular reflex test; (7) Cerebrospinal fluid neurofilament light chain. **No statistically significant difference was observed in phenotype distribution (Fisher's exact test, p=0.376) and follow-up duration (Mann-Whitney test, p=0.996) between participants showing congruent (Type2) and unexpected results (i.e., negative, Positive-Undetermined and Positive-LBD-like) with Amprion-SynSAA. ***Include reduced uptake on cardiac ¹²³I-MIBG scintigraphy, cognitive impairment, and classical pill-rolling tremor. Legend: SynSAA = α-synuclein seed amplification assay, C = cerebellar, P = parkinsonism, M = male, F = female.

**Table S3**. Level of diagnostic certainly, demographic and clinical features of PSP patients showing discrepant results between ISNB- and Amprion-SynSAA assays.

| **ID** | **Level of certainty of PSP diagnosis at last follow-up*** | **Phenotype** | **Sex** | **Age at onset (years)** | **Time onset-lumbar puncture**  **(years)** | **Time onset-last follow-up**  **(years) **** | **MRI findings** | **Less frequent features for PSP***** | **ISNB-SynSAA result** | **Amprion-SynSAA result** |
| --- | --- | --- | --- | --- | --- | --- | --- | --- | --- | --- |
| ***Discrepant SynSAA results*** | | | | | | | | | | |
| PSP-21 | probable | PSP-RS | M | 66 | 6 | 8 | IF1 | None | Negative | Positive-MSA-like |
| PSP-23 | probable | PSP-F | M | 49 | 2 | 5 | IF1 | None | Negative | Positive-MSA-like |
| PSP-27 | possible | PSP-CBS | F | 72 | 5 | 6 | IF1 | None | Negative | Positive-LBD-like |
| PSP-28 | possible | PSP-CBS | M | 73 | 5 | 8 | n/a | None | Negative | Inconclusive |
| PSP-32 | probable | PSP-RS | M | 74 | 4 | 5 | IF1 | None | Negative | Positive-MSA-like |

*According to Höglinger et al. 2017 *Movement Disorder* (ref. 3 in the main text). IF1: Atrophy is predominant in the midbrain relative to the pons. **No statistically significant difference in follow-up duration was observed between participants showing congruent (negative) and unexpected results (i.e. Inconclusive, Positive-MSA-like and Positive-LBD-like) with the Amprion-SynSAA (Mann-Whitney test, p=0.999). ***Include RBD, asymmetric parkinsonism and levodopa responsiveness. Legend: SynSAA = α-synuclein seed amplification assay, PSP-RS = PSP with Richardson’s syndrome, PSP-F = PSP with predominant frontal presentation, PSP-CBS = PSP with predominant corticobasal syndrome, M = male, F = female, MRI = magnetic resonance imaging, n/a = not available

**Table S4**. Diagnostic investigations in MSA cases with positive or discordant SAA results.

| **Diagnostic investigation** | **Positive (MSA-like)** | | **Discordant*** | |
| --- | --- | --- | --- | --- |
|  | n=101 | % | n=13 | % |
| Brain magnetic resonance imaging | 91 | 90.1 | 12 | 92.3 |
| Cerebral 123I-ioflupane-SPECT | 76 | 75.2 | 12 | 92.3 |
| Cardiac 123I-MIBG-scintigraphy | 54 | 53.5 | 5 | 38.5 |
| Neuropsychological tests | 63 | 62.4 | 12 | 92.3 |
| Videopolysomnography | 88 | 87.1 | 12 | 92.3 |
| Cardiovascular reflex test | 90 | 89.1 | 13 | 100 |
| Cerebrospinal fluid neurofilament light chain | 101 | 100 | 13 | 100 |

*Includes negative, positive-LBD-like, and positive-undetermined results
