# Supplementary material for: Comparison of Two α‐Synuclein Seed Amplification Assays for Discrimination of Parkinson Disease and Atypical Parkinsonism
Source: Mov Disord. 2025 Aug 20;40(11):2504–9. doi: 10.1002/mds.70017 (PMC12661618; doi:10.1002/mds.70017)
Supplement: Supplementary file 3 — Table S5. Comparison of the results with the two assays in each diagnostic group and participant. [file MDS-40-2504-s001.docx]

**Table S5. Comparison of the results with the two assays in each diagnostic group and participant.**

| ID | Result ISNB-SAA | Result Amprion-SAA |
| --- | --- | --- |
| MSA-1 | Negative | Positive-MSA-like |
| MSA-2 | Negative | Positive-MSA-like |
| MSA-3 | Negative | Positive-MSA-like |
| MSA-4 | Negative | Positive-MSA-like |
| MSA-5 | Negative | Positive-MSA-like |
| MSA-6 | Negative | Positive-MSA-like |
| MSA-7 | Negative | Positive-MSA-like |
| MSA-8 | Negative | Positive-MSA-like |
| MSA-9 | Negative | Positive-MSA-like |
| MSA-10 | Negative | Positive-MSA-like |
| MSA-11 | Negative | Positive-MSA-like |
| MSA-12 | Negative | Positive-MSA-like |
| MSA-13 | Negative | Positive-MSA-like |
| MSA-14 | Negative | Positive-MSA-like |
| MSA-15 | Negative | Positive-MSA-like |
| MSA-16 | Negative | Positive-MSA-like |
| MSA-17 | Negative | Positive-LBD-like |
| MSA-18 | Negative | Negative |
| MSA-19 | Negative | Positive-MSA-like |
| MSA-20 | Negative | Negative |
| MSA-21 | Negative | Positive-MSA-like |
| MSA-22 | Negative | Positive-MSA-like |
| MSA-23 | Negative | Positive-MSA-like |
| MSA-24 | Negative | Positive-MSA-like |
| MSA-25 | Negative | Positive-MSA-like |
| MSA-26 | Negative | Positive-MSA-like |
| MSA-27 | Negative | Positive-MSA-like |
| MSA-28 | Negative | Positive-MSA-like |
| MSA-29 | Negative | Positive-MSA-like |
| MSA-30 | Negative | Positive-MSA-like |
| MSA-31 | Negative | Negative |
| MSA-32 | Negative | Positive-MSA-like |
| MSA-33 | Negative | Positive-MSA-like |
| MSA-34 | Negative | Positive-MSA-like |
| MSA-35 | Negative | Positive-MSA-like |
| MSA-36 | Negative | Positive-MSA-like |
| MSA-37 | Negative | Positive-MSA-like |
| MSA-38 | Negative | Positive-MSA-like |
| MSA-39 | Negative | Positive-MSA-like |
| MSA-40 | Negative | Positive-MSA-like |
| MSA-41 | Negative | Negative |
| MSA-42 | Negative | Positive-MSA-like |
| MSA-43 | Negative | Positive-MSA-like |
| MSA-44 | Negative | Positive-MSA-like |
| MSA-45 | Negative | Positive-MSA-like |
| MSA-46 | Negative | Positive-MSA-like |
| MSA-47 | Negative | Positive-MSA-like |
| MSA-48 | Negative | Positive-MSA-like |
| MSA-49 | Negative | Positive-MSA-like |
| MSA-50 | Negative | Positive-MSA-like |
| MSA-51 | Negative | Positive-MSA-like |
| MSA-52 | Negative | Positive-MSA-like |
| MSA-53 | Negative | Positive-MSA-like |
| MSA-54 | Negative | Positive-MSA-like |
| MSA-55 | Negative | Positive-MSA-like |
| MSA-56 | Negative | Positive-MSA-like |
| MSA-57 | Negative | Positive-MSA-like |
| MSA-58 | Negative | Positive-MSA-like |
| MSA-59 | Negative | Positive-MSA-like |
| MSA-60 | Negative | Positive-MSA-like |
| MSA-61 | Negative | Negative |
| MSA-62 | Negative | Positive-MSA-like |
| MSA-63 | Positive | Positive-MSA-like |
| MSA-64 | Negative | Positive-MSA-like |
| MSA-65 | Negative | Positive-MSA-like |
| MSA-66 | Negative | Positive-MSA-like |
| MSA-67 | Negative | Positive-MSA-like |
| MSA-68 | Negative | Positive-MSA-like |
| MSA-69 | Negative | Positive-MSA-like |
| MSA-70 | Negative | Positive-MSA-like |
| MSA-71 | Positive | Positive-Undetermined |
| MSA-72 | Negative | Positive-MSA-like |
| MSA-73 | Negative | Positive-MSA-like |
| MSA-74 | Negative | Positive-MSA-like |
| MSA-75 | Negative | Positive-MSA-like |
| MSA-76 | Negative | Positive-MSA-like |
| MSA-77 | Negative | Positive-MSA-like |
| MSA-78 | Negative | Positive-MSA-like |
| MSA-79 | Negative | Positive-MSA-like |
| MSA-80 | Negative | Positive-MSA-like |
| MSA-81 | Negative | Positive-MSA-like |
| MSA-82 | Negative | Positive-MSA-like |
| MSA-83 | Negative | Positive-MSA-like |
| MSA-84 | Negative | Negative |
| MSA-85 | Negative | Positive-MSA-like |
| MSA-86 | Positive | Positive-LBD-like |
| MSA-87 | Negative | Positive-MSA-like |
| MSA-88 | Negative | Positive-MSA-like |
| MSA-89 | Negative | Positive-MSA-like |
| MSA-90 | Negative | Positive-MSA-like |
| MSA-91 | Negative | Positive-MSA-like |
| MSA-92 | Negative | Positive-MSA-like |
| MSA-93 | Negative | Positive-MSA-like |
| MSA-94 | Negative | Positive-MSA-like |
| MSA-95 | Negative | Positive-MSA-like |
| MSA-96 | Negative | Negative |
| MSA-97 | Negative | Positive-MSA-like |
| MSA-98 | Negative | Positive-MSA-like |
| MSA-99 | Negative | Positive-MSA-like |
| MSA-100 | Negative | Negative |
| MSA-101 | Negative | Positive-MSA-like |
| MSA-102 | Negative | Positive-MSA-like |
| MSA-103 | Negative | Positive-MSA-like |
| MSA-104 | Negative | Positive-MSA-like |
| MSA-105 | Negative | Positive-MSA-like |
| MSA-106 | Negative | Positive-MSA-like |
| MSA-107 | Negative | Positive-MSA-like |
| MSA-108 | Negative | Positive-MSA-like |
| MSA-109 | Negative | Positive-MSA-like |
| MSA-110 | Negative | Negative |
| MSA-111 | Positive | Positive-MSA-like |
| MSA-112 | Negative | Positive-LBD-like |
| MSA-113 | Negative | Positive-MSA-like |
| MSA-114 | Negative | Positive-MSA-like |
| PD-1 | Negative | Positive-MSA-like |
| PD-2 | Negative | Positive-LBD-like |
| PD-3 | Positive | Positive-Undetermined |
| PD-4 | Positive | Positive-LBD-like |
| PD-5 | Positive | Positive-LBD-like |
| PD-6 | Positive | Positive-LBD-like |
| PD-7 | Positive | Positive-LBD-like |
| PD-8 | Positive | Positive-LBD-like |
| PD-9 | Positive | Positive-LBD-like |
| PD-10 | Positive | Positive-LBD-like |
| PD-11 | Positive | Positive-LBD-like |
| PD-12 | Positive | Positive-LBD-like |
| PD-13 | Positive | Positive-LBD-like |
| PD-14 | Positive | Positive-MSA-like |
| PD-15 | Positive | Positive-LBD-like |
| PD-16 | Positive | Positive-LBD-like |
| PD-17 | Positive | Positive-LBD-like |
| PD-18 | Positive | Positive-LBD-like |
| PD-19 | Positive | Positive-LBD-like |
| PD-20 | Positive | Positive-LBD-like |
| PD-21 | Positive | Positive-LBD-like |
| PD-22 | Negative | Positive-LBD-like |
| PD-23 | Negative | Negative |
| PD-24 | Positive | Positive-LBD-like |
| PD-25 | Negative | Negative |
| PD-26 | Negative | Negative |
| PD-27 | Positive | Positive-LBD-like |
| PD-28 | Negative | Positive-LBD-like |
| PD-29 | Positive | Positive-LBD-like |
| PD-30 | Positive | Positive-LBD-like |
| PD-31 | Positive | Positive-LBD-like |
| PD-32 | Positive | Positive-LBD-like |
| PD-33 | Positive | Positive-LBD-like |
| PD-34 | Positive | Positive-LBD-like |
| PD-35 | Negative | Positive-LBD-like |
| PD-36 | Positive | Positive-Undetermined |
| PD-37 | Positive | Positive-LBD-like |
| PD-38 | Negative | Negative |
| PD-39 | Positive | Positive-LBD-like |
| PD-40 | Positive | Positive-LBD-like |
| PD-41 | Positive | Positive-LBD-like |
| PD-42 | Positive | Positive-Undetermined |
| PD-43 | Positive | Positive-LBD-like |
| PD-44 | Positive | Positive-LBD-like |
| PD-45 | Positive | Positive-LBD-like |
| PD-46 | Positive | Positive-LBD-like |
| PD-47 | Positive | Positive-LBD-like |
| PD-48 | Positive | Positive-LBD-like |
| PD-49 | Positive | Positive-MSA-like |
| PSP-1 | Negative | Negative |
| PSP-2 | Negative | Negative |
| PSP-3 | Negative | Negative |
| PSP-4 | Negative | Negative |
| PSP-5 | Negative | Negative |
| PSP-6 | Negative | Negative |
| PSP-7 | Negative | Negative |
| PSP-8 | Negative | Negative |
| PSP-9 | Negative | Negative |
| PSP-10 | Negative | Negative |
| PSP-11 | Negative | Negative |
| PSP-12 | Negative | Negative |
| PSP-13 | Negative | Negative |
| PSP-14 | Negative | Negative |
| PSP-15 | Negative | Negative |
| PSP-16 | Negative | Negative |
| PSP-17 | Negative | Negative |
| PSP-18 | Negative | Negative |
| PSP-19 | Negative | Negative |
| PSP-20 | Negative | Negative |
| PSP-21 | Negative | Positive-MSA-like |
| PSP-22 | Negative | Negative |
| PSP-23 | Negative | Positive-MSA-like |
| PSP-24 | Negative | Negative |
| PSP-25 | Negative | Negative |
| PSP-26 | Negative | Negative |
| PSP-27 | Negative | Positive-LBD-like |
| PSP-28 | Negative | Inconclusive |
| PSP-29 | Negative | Negative |
| PSP-30 | Negative | Negative |
| PSP-31 | Negative | Negative |
| PSP-32 | Negative | Positive-MSA-like |
| PSP-33 | Negative | Negative |
| PSP-34 | Negative | Negative |
| PSP-35 | Negative | Negative |
| PSP-36 | Negative | Negative |
| PSP-37 | Negative | Negative |
| PSP-38 | Negative | Negative |
| PSP-39 | Negative | Negative |
| PSP-40 | Negative | Negative |
| CTRL-1 | Negative | Negative |
| CTRL-2 | Negative | Negative |
| CTRL-3 | Negative | Negative |
| CTRL-4 | Negative | Negative |
| CTRL-5 | Negative | Negative |
| CTRL-6 | Negative | Negative |
| CTRL-7 | Negative | Negative |
| CTRL-8 | Negative | Positive-MSA-like |
| CTRL-9 | Negative | Positive-MSA-like |
| CTRL-10 | Negative | Negative |
| CTRL-11 | Negative | Negative |
| CTRL-12 | Negative | Negative |
| CTRL-13 | Negative | Negative |
| CTRL-14 | Negative | Negative |
| CTRL-15 | Negative | Negative |
| CTRL-16 | Negative | Negative |
| CTRL-17 | Negative | Negative |
| CTRL-18 | Negative | Negative |
| CTRL-19 | Negative | Negative |
| CTRL-20 | Negative | Negative |
| CTRL-21 | Negative | Negative |
| CTRL-22 | Negative | Negative |
| CTRL-23 | Negative | Negative |
| CTRL-24 | Negative | Negative |
| CTRL-25 | Negative | Negative |
| CTRL-26 | Negative | Negative |
| CTRL-27 | Negative | Negative |
| CTRL-28 | Negative | Negative |
| CTRL-29 | Negative | Negative |
| CTRL-30 | Negative | Negative |
| CTRL-31 | Negative | Negative |
| CTRL-32 | Negative | Negative |
| CTRL-33 | Negative | Negative |
| CTRL-34 | Negative | Positive-MSA-like |
| CTRL-35 | Negative | Negative |
| CTRL-36 | Negative | Negative |
| CTRL-37 | Negative | Negative |
| CTRL-38 | Negative | Negative |
| CTRL-39 | Negative | Positive-MSA-like |
| CTRL-40 | Negative | Negative |
| CTRL-41 | Negative | Negative |
| CTRL-42 | Negative | Negative |
| CTRL-43 | Negative | Negative |
| CTRL-44 | Negative | Positive-MSA-like |
| CTRL-45 | Negative | Positive-MSA-like |
| CTRL-46 | Negative | Negative |
